# Chronic pain trials often exclude people with comorbid depressive symptoms: A secondary analysis of 346 randomized controlled trials

**DOI:** 10.1177/17407745231182010

**Published:** 2023-06-22

**Authors:** Darren K Cheng, Maarij Hannan Ullah, Henry Gage, Rahim Moineddin, Abhimanyu Sud

**Affiliations:** 1 Lunenfeld-Tanenbaum Research Institute, Sinai Health, Toronto, ON, Canada; 2 Institute of Medical Science, Temerty Faculty of Medicine, University of Toronto, Toronto, ON, Canada; 3 Faculty of Science, McMaster University, Hamilton, ON, Canada; 4 Department of Family & Community Medicine, Temerty Faculty of Medicine, University of Toronto, Toronto, ON, Canada; 5 Humber River Hospital, Toronto, ON, Canada

**Keywords:** Chronic pain, depression, clinical trials, study populations, comorbidity, fibromyalgia, arthritis, axial pain

## Abstract

**Background:**

Chronic pain and depression are common comorbid conditions, but there is limited evidence-based guidance for management of the two conditions together. In recent years, there has been an increase in the number of chronic pain randomized controlled trials that collect depression outcomes, but it is unknown how often these trials include people with depression or significant depressive symptoms. If trials do not include participants representative of real-world populations, evidence and guidance generated from these trials risk being inapplicable for large proportions of the target population, or worse, risk harm. Thus, in order to identify pathways to improve the conduct of clinical trials, the aims of this study were to (1) estimate the proportion of randomized controlled trials evaluating chronic pain interventions and reporting depression outcomes that include participants with significant depressive symptoms; and (2) assess the variability of inclusion proportions by pain type, intervention type, gender, country of origin, and publication year.

**Methods:**

Studies were extracted from an umbrella review of interventions for chronic pain that reported depression outcomes. Screening and data extraction were completed in duplicate and conflicts were resolved by a third author. Randomized controlled trials with at least 50% adult participants and validated depression scales were included, and randomized controlled trials with populations whose mean scores were at or above depression thresholds at baseline were considered to have included participants with depression.

**Results:**

Of the 346 randomized controlled trials analyzed, 142 (41%) included participants with depression. Eight pain-type groups and nine intervention types were identified. Randomized controlled trials investigating fibromyalgia and mixed chronic pain had the highest proportion of participants with depression, whereas studies of arthritis and axial pain had among the lowest. Randomized controlled trials from the United States had a significantly lower inclusion proportion compared with non-US studies, especially for studies on arthritis. The increase in inclusion proportion by publication year was driven by the increase in fibromyalgia studies.

**Discussion and Conclusion:**

This study highlights opportunities to improve the conduct of chronic pain clinical trials. The majority of randomized controlled trials s analyzed evaluated participants without significant depressive symptoms at baseline, thus the findings synthesized in systematic reviews and subsequent guidelines are most applicable to the subset of real-world populations that do not have significant depressive symptoms. As well, systemic biases around psychological conditions and gender may be important contributors to differences in the study of depression in fibromyalgia compared with common conditions such as arthritis and axial pain. In order to better inform clinical practice, future research must intentionally include individuals with comorbid depression in trials of common chronic pain conditions, and consider methods to mitigate biases that may distort study design.

## Background

Approximately 20% of North American adults live with chronic pain^1,2^—a disabling group of health conditions associated with a number of mental illness comorbidities, including major depressive disorder.^3^ Estimates of comorbid chronic pain and major depressive disorder range from 27% in primary care to 85% in other specialty settings,^4^ well above the prevalence of 8%–10% in the general population.^5,6^ This comorbidity is associated with poorer well-being and functionality compared with chronic pain alone,^7,8^ including lower mental and physical quality of life,^7,9^ increased intensity of pain, and reduced pain tolerance.^10,11^ Chronic pain has also been found to increase the frequency and duration of depressive episodes.^12^ Interest in chronic pain treatments and research has increased substantially over the past decades, with concomitant calls and commitments for dedicated funding in multiple jurisdictions.^13,14^ Addressing this comorbidity using relevant evidence should be considered a high clinical and policy priority.

Unfortunately, there is minimal evidence-based guidance on the appropriate management of major depressive disorder in chronic pain. As one example, a national guideline for chronic pain management included a single recommendation for the management of major depressive disorder, which cited only one randomized controlled trial (RCT).^15^ In recent years, the number of chronic pain trials that report depression outcomes has increased.^16^ However, the proportion of these studies that include participants with significant depressive symptoms at baseline is unknown. While the research on this topic is still nascent, it has been recently identified that eligibility criteria in chronic pain trials are overly restrictive and often exclude highly prevalent mental illnesses.^17^ This aligns with findings that people living with mental illnesses, such as major depressive disorder, are systematically excluded from clinical trials of chronic conditions, including chronic pain, chronic obstructive pulmonary disease, and diabetes.^18,19^ Mental illness comorbidities, and multi-morbidity in general, are often characterized as confounders, and the resulting exclusion of people living with mental illness comorbidities substantially limits the external validity of these trials to real-world populations.^17,19^ Excluding those with comorbid mental illness from chronic pain trials may increase risk of harm as the research guiding clinical practice will not sufficiently capture how these individuals respond to interventions.^19^ It is important to investigate trials and their characteristics to better understand where biases in trial design may arise, and how this may have changed over time. Therefore, in order to identify pathways for improving the real-world applicability of chronic pain trials, in this study we aimed to (1) estimate the proportion of RCTs evaluating chronic pain interventions and reporting depression outcomes that have included participants with significant depressive symptoms at baseline; and (2) assess the variability of inclusion proportions by important factors that may impact RCT design and conduct including pain type, intervention type, gender, country of origin, and publication year.^20–24^

## Methods

We took a novel and opportunistic approach to addressing our research objectives by conducting a secondary analysis of a systematic review of systematic reviews (or umbrella review) of interventions for chronic pain that reported depression outcomes.^16^ Examining this evidence base provides an important perspective on the populations captured in RCTs and synthesized by systematic reviews which are ultimately used to inform the development of clinical practice guidance and policy. We identified all RCTs with a depression outcome that were included in any of the systematic reviews included in the umbrella review. We determined inclusion of participants with significant depressive symptoms based on the available baseline characteristics of trial participants. Given the available data from published reports of RCTs, we determined the inclusion proportion at the trial level rather than at the individual patient level.

### Information sources

The umbrella review included systematic reviews which (1) studied any intervention for chronic pain and (2) reported aggregate scores of depression outcome measures.^16^ All RCTs included in analyses of the 67 reviews from the umbrella review were considered for this study.

### Eligibility criteria

To be eligible for inclusion, studies had to (1) use an RCT design evaluating efficacy or effectiveness; (2) include a study sample of at least 50% adults (18 years or older) experiencing chronic pain (as per the International Association for the Study of Pain definitions,^25^ chronic pain was interpreted as pain in any body part lasting for a least 3 months), excluding populations exclusively living with cancer and/or end-of-life pain; and (3) use a validated depression scale, which included, but was not limited to, the Beck Depression Inventory (BDI-I and BDI-II), Hospital Anxiety and Depression Scale (HADS), Hamilton Depression Rating Scale (HAM-D), Center for Epidemiological Studies Depression Scale (CES-D), Montgomery–Asberg Depression Rating Scale (MADRS), Patient Health Questionnaire-9 (PHQ-9), and Geriatric Depression Scale (GDS) (Table 1). While there are overlaps in the pathogenesis of chronic cancer pain and chronic noncancer pain, these groups of conditions are typically studied separately and different treatment approaches and pathways are deployed for management.^26^ Thus, this study is focused specifically on trials for chronic noncancer pain.

**Table 1.** Minimum depression scores for validated outcome measures.

Depression scale	Minimum score classified as depressed^a^
Beck Depression Inventory I (BDI-I)	10^27^
Beck Depression Inventory II (BDI-II)	14^28^
Beck Depression Inventory—short form (BDI-SF)	5^29^
Hospital Anxiety and Depression Scale (HADS-D)	8^28^
Hamilton Rating Scale for Depression/Hamilton Depression Rating Scale (HAM-D or HRSD/HDRS)	8^30^
Center for Epidemiologic Studies Depression Scale (CES-D)	19^31^
10-item CES-D (CES-D-10)	10^32^
Chicago Multiscale Depression Inventory (CMDI)	81 (total); 23 (mood); 22 (evaluative)^33^
Montgomery–Asberg Depression Rating Scale (MADRS)	7^34^
Patient Health Questionnaire (9-item; PHQ-9)	10^28^
Patient Health Questionnaire (8-item; PHQ-8)	10^35^
Geriatric Depression Scale (GDS)	5 (short form), 11 (long form)^28^
Depression Anxiety Stress Scales (DASS-21-D)	10^36^
Comprehensive Psychopathological Rating Scale (CPRS-A)	1.0^37^

a Minimum scores were identified in studies on chronic pain populations whenever possible.

Studies of any clinical intervention that addressed chronic pain and its related symptoms were eligible for inclusion. Expected interventions included, but were not limited to pharmacological (such as NSAIDs, opioids, and antidepressants), physical (such as physical therapy and massage), and psychological (such as cognitive behavioral therapy or acceptance and commitment therapy) interventions. RCTs of any duration and context, and any comparator type were eligible. Due to resource constraints, only English language studies were included.

### Study selection and data extraction

We screened systematic reviews from the umbrella review for potential primary articles by examining “Study Characteristics” tables, “Description of Included Studies” sections, primary results sections, and reference lists. Two authors completed this process independently and in duplicate. After removing duplicate records from this screening stage, full-text articles were retrieved. Two authors screened all full-text articles independently and in duplicate for the eligibility criteria listed above. A third author resolved conflicts from both stages of study selection.

From included articles, two independent reviewers extracted in duplicate the following data items: first author, publication year, country of origin, chronic pain type, intervention type, participant gender distribution, and depression outcome measure. We examined whether the baseline population met thresholds for significant depressive symptoms by comparing the mean baseline depression score for RCT participants (pooled across intervention and control arms) with commonly cited minimum thresholds (Table 1). We dichotomously categorized RCTs with mean scores above thresholds as having *included participants* with significant depressive symptoms and those with subthreshold mean scores as having *excluded* them. A third author resolved conflicts in data extraction.

Our primary outcome was the proportion of RCTs that included participants with significant depressive symptoms, which is reported as “inclusion proportion” for categories of RCTs. We used Fisher’s exact tests to assess the association between inclusion proportion and chronic pain type, intervention type, country, and over time. We reported odds ratios (OR) and 95% confidence intervals (CI) for the likelihood of women participants at baseline. Given the high proportion of fibromyalgia studies and their high inclusion proportion, all analyses were stratified with and without fibromyalgia studies. We used SAS 9.4 for statistical analysis. All tests were two-sided and p < .05 was considered statistically significant.

## Results

**Figure 1.**
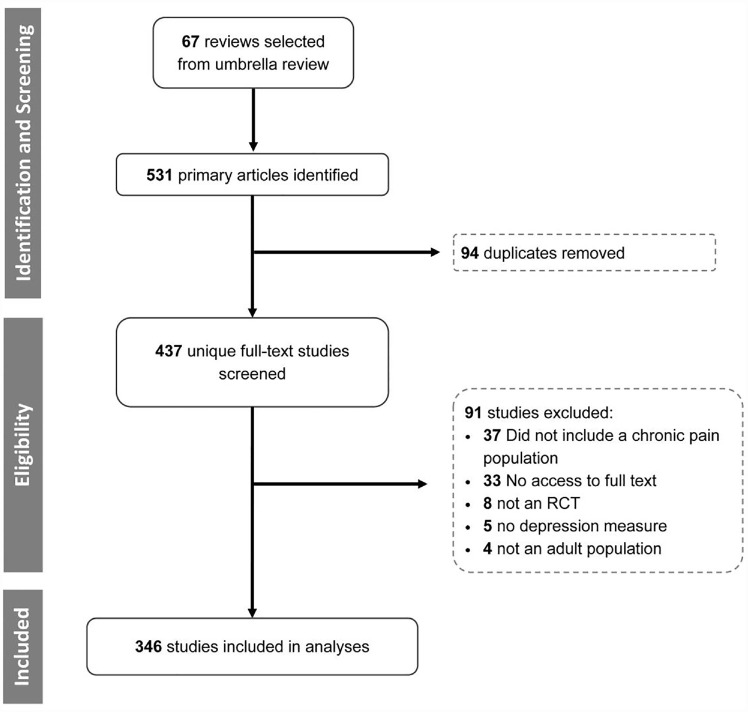
Flow diagram.

A total of 346 RCTs, published between 1981 and 2018, were included in the analysis (see Figure 1 for reasons for exclusion). Of the included RCTs, 142 (41.0%) included participants with significant depressive symptoms at baseline. The remaining 204 RCTs included participants with subthreshold depressive symptoms (n = 114), used non-validated measures (n = 80), or did not report baseline depression scores (n = 10).

We identified eight distinct chronic pain types and nine intervention types based on RCT descriptions (Supplemental Material). Fibromyalgia arthritis and axial pain were the most common pain types and psychological, pharmacological, and exercise were the most common intervention types.

### Pain types

Inclusion proportion was significantly different across pain types with mixed chronic pain and fibromyalgia RCTs having the highest (61.1% and 57.1%, respectively) and arthritis and axial pain RCTs having among the lowest (18.6% and 26.9%, respectively) (Table 2).

**Table 2.** Inclusion proportion of RCTs by chronic pain type, and intervention type (stratified by fibromyalgia studies).

Groups	Classifications	All studies		Excluding fibromyalgia studies	
		Inclusion proportion (number of trials including people with depression/total number of trials)	p value	Inclusion proportion (n/total n)	p value
All RCTs	–	41.0% (142/346)	–	29.1% (58/199)	–
Chronic pain type	*Fibromyalgia*	57.1% (84/147)	**<.001**	–	–
	*Arthritis*	18.6% (13/70)		18.6% (13/70)	**.001**
	*Axial*	26.9% (14/52)		26.9% (14/52)	
	*Mixed*	61.1% (22/36)		61.1% (22/36)	
	*Neuropathic*	20.0% (3/15)		20.0% (3/15)	
	*Orofacial*	21.4% (3/14)		21.4% (3/14)	
	*Musculoskeletal*	40.0% (3/5)		40.0% (3/5)	
	*Headache*	0% (0/3)		0% (0/3)	
	*Unclassified*	25.0% (1/4)		25.0% (1/4)	
Intervention type^a^	*Psychological*	38.4% (48/125)	**.009**	33.3% (32/96)	.101
	*Pharmacological*	53.4% (39/73)		29.2% (7/24)	
	*Exercise*	40.3% (25/62)		9.5% (2/21)	
	*Physical*	37.1% (13/35)		38.9% (7/18)	
	*Education*	20.6% (7/34)		17.9% (5/28)	
	*Mind-body*	36.7% (11/30)		16.7% (3/18)	
	*Web/telephone-based*	54.5% (12/22)		52.9% (9/17)	
	*Technological*	73.7% (14/19)		33.3% (1/3)	
	*Multi-disciplinary*	30.0% (3/10)		22.2% (2/9)	
	*Other*	40.0% (4/10)		40.0% (4/10)	

Statistically significant p values (<.05) are given in bold.

a Intervention types are non-cumulative.

After removing the fibromyalgia studies, the inclusion proportion for the remaining 199 RCTs dropped to 29.1% and there was still a significant difference across pain types.

### Intervention types

Inclusion proportion was significantly different across intervention types, with RCTs investigating technological (e.g. repetitive transcranial magnetic stimulation), web-based, and pharmacological interventions having the highest inclusion proportions (73.7%, 54.5%, 53.4%, respectively), and greater than the mean across the 346 RCTs (Table 2).

After excluding fibromyalgia studies, differences in inclusion proportions across intervention types were no longer significant (Table 2). Inclusion proportions without fibromyalgia dropped the most for technological, pharmacological, and exercise interventions.

### Gender

RCTs that had 90%–100% women participants were 2.6 times more likely to include participants with depression than RCTs that had 0%–49% women participants, but this effect was no longer significant when fibromyalgia studies were removed (Table 3). There was no significant difference in inclusion proportion between RCTs with 50%–90% women participants compared with 0%–49% both with and without fibromyalgia.

**Table 3.** Inclusion proportion of RCTs by percentage women participants (stratified by fibromyalgia studies).

Groups	Classifications	All studies		Excluding fibromyalgia studies	
		Inclusion proportion (n/total n)	OR (95% CI)	Inclusion proportion (n/total n)	OR (95% CI)
Percentage of women participants^a^	*0% to <50%*	32.4% (11/34)	–	31.3% (10/32)	–
	*50%*≤*to <90%*	30.8% (48/156)	0.93 (0.42–2.06)	29.0% (42/145)	0.90 (0.39–2.06)
	*90%*≤*to 100%*	55.7% (78,140)	**2.63** (1.19–5.81)	27.3% (6/22)	1.22 (0.33–4.60)

Statistically significant OR (95% CI) are given in bold.

a Of 346 RCTs, 330 reported gender at baseline; OR (95% CI) are compared with 0% to <50% group.

### Country of origin

Articles were published from 28 different countries, with the relative majority from the United States (US; n = 139). The inclusion proportion of US RCTs was 31.7%, which was significantly lower than the proportion for all non-US RCTs of 47.3% (Table 4). When excluding fibromyalgia RCTs, the inclusion proportion dropped and the difference between US and non-US RCTs was even more apparent (18.9% and 37.6%).

**Table 4.** Inclusion proportion by country stratified by fibromyalgia studies, and by country and pain type.

	US		Non-US	
All RCTs	Inclusion proportion (n/total n)		Inclusion proportion (n/total n)	p value
With fibromyalgia	31.7% (44/139)		47.3% (98/207)	**.004**
Without fibromyalgia	18.9% (17/90)		37.6% (41/109)	**.004**
By pain type	Inclusion proportion (n/total n)	p value	Inclusion proportion (n/total n)	p value
Fibromyalgia	55.1% (27/49)	**<.001**	58.2% (57/98)	**.005**
Arthritis	9.8% (4/41)		31.0% (9/29)	
Axial	18.8% (3/16)		30.6% (11/36)	
Mixed	60.0% (6/10)		61.5% (16/26)	
Neuropathic	14.3% (1/7)		25.0% (2/8)	
Orofacial	25.0% (3/12)		0.0% (0/2)	
Headache	0.0% (0/2)		0.0% (0/2)	
Musculoskeletal	0.0% (0.2)		66.7% (2/3)	
Unclassified	0.0% (0/1)		33.3% (1/3)	

Statistically significant p values (<.05) are given in bold.

Among the US RCTs, arthritis RCTs were the second-most common pain type and had the lowest inclusion proportion of any pain type at 9.8%. The comparable inclusion proportion for non-US arthritis studies was 31.0% (Table 4).

### Publication year

**Figure 2.**
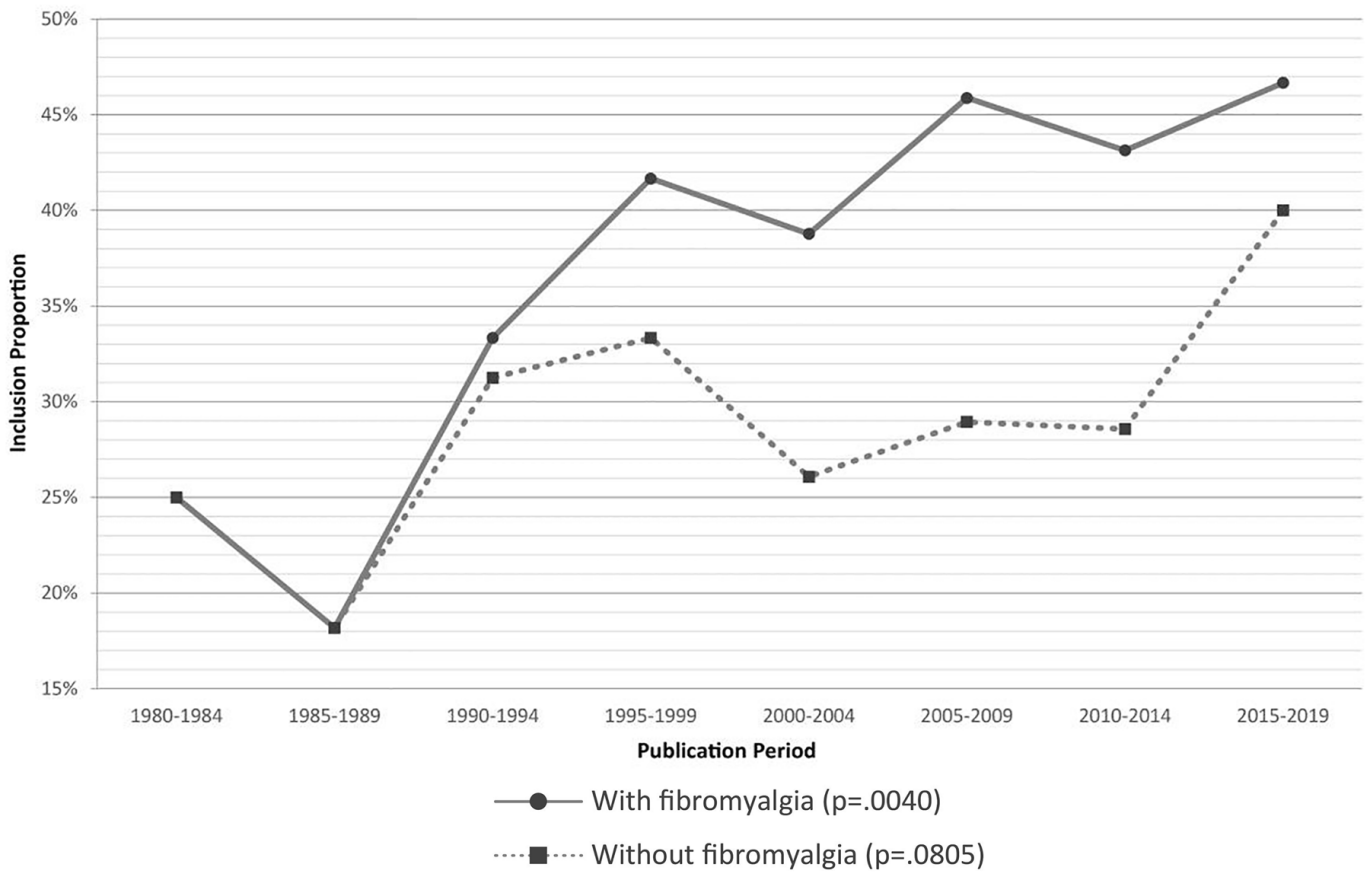
Overall inclusion proportion over time (stratified by fibromyalgia studies).

The inclusion proportion significantly increased by 3.7% per 5-year publication period (Figure 2). When excluding fibromyalgia RCTs, the increase in inclusion proportion decreased to 1.7% per 5-year publication period and was no longer significant.

## Discussion

### Summary of findings

From a large, systematically derived corpus spanning four decades, we determined that 59% of RCTs for chronic pain that report depression outcomes did not include participants with significant depressive symptoms at baseline. This proportion is likely an underestimate for a more widespread issue as RCTs that do not report depression outcomes were not captured in this study and may be more likely to exclude participants with significant depressive symptoms.

The inclusion proportion varied significantly by pain type, intervention type, by percentage of women participants, country of origin, and over time. For common pain types such as arthritis and axial pain, inclusion proportions were as low as 19% and 27%, respectively. The significant variations in intervention type, gender, and over time, however, were driven by the large number of recent fibromyalgia studies with much higher inclusion proportions. For intervention types, the high inclusion proportion in RCTs of technological interventions such as repetitive transcranial magnetic stimulation, may be explained by their frequent application for treatment-resistant depression.^38^

Overall, improvements in inclusion proportions over the past four decades may be illusory, representing an increase in fibromyalgia studies and their high inclusion proportion, rather than a growing interest in including participants with significant depressive symptoms across all chronic pain conditions. This timing aligns with when fibromyalgia diagnosis criteria were first developed and promulgated in the 1980s.^39^ In addition, a relative majority of RCTs originated from the United States, but this set had a lower inclusion proportion compared with other countries, primarily driven by low inclusion proportions in arthritis studies.

### Interpretations

This secondary analysis of 346 RCTs over four decades and 18 countries provides a high-level review of the chronic pain evidence landscape as it pertains to significant depressive symptoms. Findings from this study highlight two critical issues regarding the design and conduct of RCTs in chronic pain and therefore the subsequent reviews and clinical guidance that may be based on these RCTs: a failure to account for psychological dimensions of living with arthritic pain, especially in the United States; and the potential distortion of current evidence due to biases regarding fibromyalgia, and sex and gender.

RCTs do not seem to adequately conceptualize the psychological dimensions of living with chronic pain by failing to include participants that are representative of real-world populations. Fibromyalgia studies were the most likely to include participants with depression, while arthritis and axial pain had some of the lowest inclusion proportions. This is despite fibromyalgia, arthritis, and axial pain all having comparable rates of comorbidity with depression.^40–45^ This differential is reflected even more starkly in the analysis by country. The inclusion proportion for US studies was significantly lower compared with all other countries combined, while making up 40.2% of the total number of RCTs analyzed in this study. This is largely driven by the large number of arthritis studies published by the United States, which had a very low inclusion proportion, even compared with arthritis studies in other countries. This is concerning, not only because of the high prevalence of arthritis, but also considering that comorbid arthritis and major depressive disorder have been found to increase functional limitations, financial burden,^46^ and mortality.^47^ Furthermore, there is currently little evidence-based clinical guidance for treatment of depression in these pain conditions; for example, one current clinical practice guideline for arthritis management recommends only a single intervention, Tai Chi, for managing depression.^15^ It appears that RCTs in the United States, as compared with research from other countries, are less likely to consider the psychological dimension of the arthritis experience and rarely capture participants that experience mental illness. This suggests that reframing conceptions of the psychological dimensions of the chronic pain experience as common and relevant, regardless of chronic pain type, is essential to generating appropriate trials evidence to guide clinical management. Future research must prioritize investigating the impact of such conceptions on treatment and real-world outcomes for depression across pain types.

There was also a predominance of fibromyalgia RCTs which also overlapped with RCTs that included largely women participants and individuals with depression. This convergence suggests that gender bias may be an important contributor to these findings, given the known long-standing perceptions, and treatment, of women living with pain as having more significant psychological rather than physical symptomatology.^48^ It may also be due to biased conceptions of fibromyalgia as having more relevant psychological dimensions than other pain conditions;^49^ biased conceptions about fibromyalgia as a disease predominantly affecting women;^50^ and the fact that women are more likely to seek treatment for psychological symptoms alongside the chronic pain comorbidity.^51^ These kinds of biases may also manifest, for example, in different standardized trial reporting measures across pain types, with fibromyalgia studies being more likely to standardize the inclusion of depression outcomes.^50^ These biases provide some potential explanations for the convergence of these study characteristics in fibromyalgia studies. Future chronic pain RCTs should prioritize explicit sex and gender based analyses that acknowledge and account for such pervasive biases at every level of the clinical trial process, from design to delivery to analysis.^52,53^

While this study was not designed to evaluate *why* participants with depression are excluded from chronic pain clinical trials, previous studies suggests multiple factors may limit recruitment.^54^ These may include stigma surrounding the admission of having mental illness,^54,55^ risk aversion,^56^ distrust in medical research especially in historically marginalized groups,^57^ and ethical dilemmas related to potential negative impact on vulnerable patients,^58^ particularly in trials with intervention arms perceived as being inferior^59,60^ or involving deception.^61^ Efforts must be made to design research trials that minimize these and other obstacles to participation for people living with comorbid chronic pain and depression.

### Strengths and limitations

In this study, we examined depression inclusion proportions over a period of four decades across pain types, intervention types, gender, and country. This large corpus, derived from a systematically developed umbrella review, allowed for the comparison of inclusion proportions across multiple categories and provided a broad view of the research landscape.

We examined RCTs captured in an umbrella review that specifically investigated systematic reviews reporting depression outcomes. The aim of this sub-analysis is distinct from explicitly investigating RCTs that intentionally included people with depression or RCTs investigating interventions for improving depressive symptoms in people with chronic pain. This provided a sampling that reflected the evidence that informs clinical guidelines and practice relevant to people living with chronic pain and depression. However, RCTs that did not evaluate or report depressive symptoms were not captured and analyzed. This would likely result in the reported inclusion proportion being an *overestimate* of the inclusion proportion across all chronic pain RCTs, many of which explicitly exclude people living with comorbid mental illness.^18,19^

Since inclusion of RCTs in this study are dependent on their inclusion in systematic reviews, there may be relevant studies, especially more recent ones, that were not included in this analysis. The reliability of the data that have been collected is bolstered by comprehensive and independent study selection and data extraction from three independent authors. However, due to the nature of available data, analyses were restricted to the trial- rather than participant-level and so smaller RCTs could have had disproportionate impacts on the inclusion proportions described. Chronic cancer pain and comorbid depression is an important clinical issue that was not addressed in this study as this literature was not reviewed. Additional analyses of RCTs including people living with chronic cancer pain may identify different trends and opportunities for improving trial conduct. The grouping of pain and intervention types was done using study descriptions and is dependent on the sometimes heterogeneous populations included in the RCTs, resulting in synthetic categories such as “musculoskeletal” pain and “multidisciplinary” interventions. Since these categories are aggregates of the ways that research studies delineate these groups, they may not fully reflect how these conditions or interventions are characterized or used in clinical practice. As identified elsewhere^16,62^ clinical trials would benefit from more clear and standardized descriptions of included interventions and populations, including definitions for types of chronic pain.

This study captures a subset of existing evidence in chronic pain research, but identifies issues that are likely to be larger and more widespread when investigated more broadly. Alternative methods of investigation could include conducting a systematic review and synthesis of all RCTs that investigate chronic pain and report depression outcomes rather than extracting from systematic reviews—this may capture a more fulsome set of existing trial evidence. As well, analyses of clinical guidelines and the RCTs that inform recommendations could provide a clearer picture of the representativeness of trial populations in guidance statements.

## Conclusion

Findings from this study highlight the importance of robust study design to enable the generation of clinically relevant and appropriate evidence for managing chronic pain and depression. In this study, we found that the majority of RCTs captured in recent systematic reviews of chronic pain interventions and reporting depressive symptoms outcomes largely report findings most applicable to people with chronic pain who are mildly or not depressed at the beginning of treatment.

Given the high prevalence of comorbid chronic pain and depression, future RCTs investigating interventions for people with chronic pain must work to include participants with depression, or at the very least, significant depressive symptoms. This could be done through attention to trial eligibility criteria and ensuring that people living with mental illness are not excluded.^17^ This is especially important for conditions like arthritis and axial pain that are often under-investigated for their psychological dimensions. Recruiting participants with significant depressive symptoms is often difficult,^54,63,64^ and so researchers should seek out partnerships with care settings and institutions that provide services for people with mental illness and/or complex conditions that co-occur with chronic pain. Furthermore, consideration must be given during study planning phases to minimize biases that may limit applicability of research findings. Researchers must carefully consider including explicit sex and gender-based analyses, and using validated outcome measures that capture both physical and psychological dimensions.

## Supplemental Material

sj-docx-1-ctj-10.1177_17407745231182010 – Supplemental material for Chronic pain trials often exclude people with comorbid depressive symptoms: A secondary analysis of 346 randomized controlled trials Supplemental material, sj-docx-1-ctj-10.1177_17407745231182010 for Chronic pain trials often exclude people with comorbid depressive symptoms: A secondary analysis of 346 randomized controlled trials by Darren K Cheng, Maarij Hannan Ullah, Henry Gage, Rahim Moineddin and Abhimanyu Sud in Clinical Trials
