# Supplementary material for: Chronic pain trials often exclude people with comorbid depressive symptoms: A secondary analysis of 346 randomized controlled trials
Source: Clin Trials. 2023 Jun 22;20(6):632–41. doi: 10.1177/17407745231182010 (PMC10638851; doi:10.1177/17407745231182010)
Supplement: sj-docx-1-ctj-10.1177_17407745231182010 – Supplemental material for Chronic pain trials often exclude people with comorbid depressive symptoms: A secondary analysis of 346 randomized controlled trials [file sj-docx-1-ctj-10.1177_17407745231182010.docx]

**Supplementary File 1. *Pain and Intervention Types and Descriptions***

| **Category** | **Description** |
| --- | --- |
| **Pain Type** | |
| ***Fibromyalgia*** | Chronic widespread musculoskeletal pain and tenderness. |
| ***Arthritis*** | Includes osteoarthritis, rheumatoid arthritis, and ankylosing spondylitis. |
| ***Axial*** | Chronic low back and neck pain of any aetiology. |
| ***Neuropathic*** | Pain caused by a lesion or disease of the somatosensory nervous system. |
| ***Orofacial*** | Pain to the mouth or face, primarily temporomandibular joint disorder. |
| ***Musculoskeletal*** | Conditions like osteoarthritis and fibromyalgia studied together and which could not be disaggregated. |
| ***Headache*** | Chronic persistent headache present ≥15 days per month for ≥3 months. |
| ***Mixed*** | Pain conditions from multiple categories above that were grouped together in RCTs. |
| ***Unclassified*** | Pain type unclear or unclassified. |
| **Intervention Type** | |
| ***Psychological*** | Psychological therapies focused on changing cognitive, behavioural, or emotional activity. |
| ***Pharmacological*** | Prescription medications, not including herbal supplements. Included primarily SSRIs, SNRIs, TCAs, and gabapentinoids. |
| ***Mind-Body*** | Interventions focused on the relationships among the brain, mind, body, and behaviour, and their effect on health and disease. Generally focused on relaxation, consciousness, attention, and/or the body (meditation) or involved exercise or movements with focused attention on breathing and movement of the body (mindful movement). |
| ***Exercise*** | Programs that primarily included aerobic exercise, flexibility, stretching, endurance, and/or strength training. |
| ***Technological*** | Non-invasive brain stimulation interventions. |
| ***Physical*** | Massage therapy that involves manipulation of the soft tissues in a systematic way - excludes Reiki and other manual therapy such as chiropractic, or spinal manipulation. |
| ***Education*** | Interventions explicitly described as education programs; formal structured instructions largely on managing chronic pain symptoms. |
| ***Web- and telephone- based*** | Psychologically-based programs or therapies delivered using non-face-to-face methods. |
| ***Multidisciplinary*** | Programs that involved components from multiple other intervention categories (e.g. psychological, education, exercise, physical therapy, self-management). |
| ***Other*** | Other interventions that did not fit into other categories. |
